# TEAMwork: Interplay of Post‐Transcriptional Mechanisms, Epigenetics and Metabolism in (Auto‐)Immunity

**DOI:** 10.1002/eji.70064

**Published:** 2025-10-08

**Authors:** Francesca Rossi, Martin Turner

**Affiliations:** ^1^ Immunology Programme, The Babraham Institute Babraham Research Campus Cambridge UK

**Keywords:** autoimmunity, epigenetics, lymphocytes, metabolism, RNA‐binding proteins

## Abstract

Changes in transcript abundance and isoforms, mediated by epigenetic and post‐transcriptional mechanisms, are a hallmark of the development, activation, and effector functions of immune cells. How epigenetic and post‐transcriptional processes are orchestrated to regulate transcription and pre‐mRNA processing, and their interplay with metabolism, is emerging as important for immunity. DNA and histone modifications recruit RNA‐binding proteins (RBPs) to mediate co‐transcriptional RNA processing at specific chromatin *loci*. Simultaneously, RBPs influence the deposition of epigenetic modifications by regulating the expression of chromatin‐modifying enzymes and enzymes that control the amounts of metabolites. These are used as substrates by chromatin‐modifying enzymes and can influence RBP activity; thus, modulation of metabolic pathways represents a mechanism to regulate the epigenetic landscape and pre‐mRNA processing. A body of work identifies emerging regulatory principles that address the interplay between epigenetics and RBPs in the nucleus, and of cytoplasmic post‐transcriptional mechanisms that regulate metabolism and epigenetics. In this review, we focus on the interconnections between RBP‐mediated processes, chromatin modifications, and metabolic pathways, highlighting the role that such circuits have in T‐ and B‐lymphocytes, and in autoimmunity.

## Graphical abstract

This review discusses the principles underlying the interplay of epigenetics, metabolism, and post‐transcriptional regulation of gene expression mediated by RNA‐binding proteins, both in the nucleus and in the cytoplasm. Particularly, it focuses on the relevance that these circuits have in lymphocytes and autoimmunity.

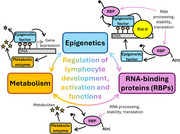

**Abbreviations**
5mC: 5‐methylcytosine
Acetyl‐CoA: Acetyl‐Coenzyme‐A
ACLY: ATP Citrate Lyase
ACSS2: Acyl‐CoA Synthetase Short‐chain family member‐2
APA: Alternative polyadenylation signal
AS: Alternative splicing
CAR: Chimeric antigen receptor
CPA: Cleavage and polyadenylation
CTCF: CCCTC‐binding factor
DRBP: DNA/RNA‐binding protein
hnRNP: Heterogeneous nuclear ribonucleoprotein
ICF: Immunodeficiency Centromeric instability and Facial anomalies
PAS: Polyadenylation signal
PDH: Pyruvate dehydrogenase
PKM: M‐type‐pyruvate kinase
Pol‐II: RNA Polymerase‐II
PSIP1: PC4‐ and SF2‐Interacting Protein‐1
PTBP1: Polypyrimidine Tract Binding Protein 1
PTM: Post‐translational modification
RBM25: RNA‐binding motif protein 25
RBP: RNA‐binding protein
SAM: S‐adenosylmethionine
SLE: Systemic lupus erythematosus
SRSF: Serine‐arginine‐rich splicing factor
TET: Ten Eleven Translocase
Th cells: T‐helper cells
Treg cells: Regulatory T‐cells
UTR: Untranslated region

## 1 Introduction

A significant body of research has identified epigenetics, post‐transcriptional mechanisms, and metabolism as key determinants in immune cell biology, in both physiological and aberrant immune responses. This review focuses on the interconnection among these processes in the adaptive immune system, highlighting how they contribute to autoimmunity. Recently, the relevance of epigenetic and metabolic regulation in innate immunity, particularly trained immunity [1], has also become apparent [2, 3, 4, 5], and a topical question is how post‐transcriptional mechanisms participate in these processes. While we recognise its importance, we will not focus on this here but point the reader to dedicated reviews [1, 2, 3, 4].

Below, we introduce the concepts of epigenetics, post‐transcriptional mechanisms and metabolism, and the therapeutic potential of their modulation in autoimmunity.

### 1.1 Epigenetics

The term epigenetics encompasses those mechanisms of gene regulation that take place at the DNA level without altering the sequence. DNA methylation (i.e., 5‐methylcytosine, 5mC), histone post‐translational modifications (PTMs), and chromatin‐interacting factors (e.g., proteins and non‐coding RNAs) establish a chromatin environment that either promotes or hinders transcription [6, 7]. Reversibility is a feature of epigenetic modifications that allows them to mediate responses to cellular signals and environmental stimuli [8]. DNA methylation and histone PTMs can be inherited along with genetic information through cell division [9], transforming a response to an environmental stimulus into a heritable feature.

### 1.2 Post‐Transcriptional Regulation of Gene Expression

Post‐transcriptional mechanisms are considered the most rapid way in which cells respond to stimuli, as in the case of translationally poised mRNAs that are quickly released to be actively translated [10], or the removal from pre‐mRNAs of destabilising “poison” exons [11, 12]. Small RNAs and RNA‐binding proteins (RBPs), which directly bind to RNA, are key regulators at every step of RNA biogenesis, translation, and degradation [13, 14]. Chemical modifications of RNA bases (e.g., N6‐methyladenosine and A‐to‐I editing), long non‐coding and circular RNAs, have also emerged as important post‐transcriptional modulators of RNA metabolism, increasing the complexity of gene expression regulation [15, 16, 17, 18].

### 1.3 Metabolism

Metabolic reactions allow cells to extract energy from nutrients as well as synthetise biomass. There is a well‐established link between metabolism and epigenetics [19]. Besides histone/DNA methylation, dependent on S‐adenosylmethionine (SAM), and histone acetylation dependent on acetyl‐coenzyme‐A (acetyl‐CoA), many more epigenetic modifications rely on metabolites (including citrullination and different forms of acylation, for example, lactylation and succinylation) [19]. Metabolites have also been shown to influence RBPs’ activity [20]. Vice versa, RBPs can target mRNAs encoding metabolic enzymes, modulating metabolite levels [21, 22, 23].

Metabolic regulation is fundamental for lymphocyte differentiation and activation [24, 25]. Given the complexity of the mechanisms involved, we would like to highlight the importance of deep validation and extensive investigation in immune‐metabolism research, through two examples. First, the suitability of metabolic assays for studying metabolism in lymphocytes needs to be tested. Recently, the 2‐(*N*‐(7‐nitrobenz‐2‐oxa‐1,3‐diazol‐4‐yl)amino)‐2‐deoxyglucose labelling assay to measure single‐cell glucose uptake was demonstrated to be unsuitable for T‐cells, as it is not blocked by glucose transporter inhibitors or excess glucose, and therefore lacks specificity for glucose transporters expressed by T‐cells [26]. Second, the effects of metabolite analogues, used to modulate metabolism, need to be carefully considered to avoid misinterpretation when they may mediate their effects through multiple mechanisms of action. This is the case of 2‐deoxy‐d‐glucose, which interferes with glycolysis and induces cytotoxicity in tumour cells, but also inhibits N‐linked glycosylation and membrane expression of tumour markers recognised by immune cells [27].

### 1.4 Therapeutic Relevance of Epigenetics, RBPs, and Metabolism Modulation in Lymphocytes

Autoimmunity is caused by a chronic overactivation of B‐ and T‐lymphocytes by self‐antigens. Genetics and environment contribute to this harmful immune response, which leads to sustained inflammation, tissue damage, and organ dysfunction [28, 29]. Most of the current treatments for autoimmune conditions involving pathway or cytokine inhibitors are not disease‐specific, induce side effects (e.g., weakening of the immune system), may not be effective in some patients, or their effectiveness decreases over time [30]. Recent successes of clinical trials using chimeric antigen receptor (CAR) T‐cell‐based therapies to treat severe autoimmune conditions are opening new therapeutic scenarios [31]. Research is now focusing on potentiating CAR‐T‐cell functions to improve treatment outcomes.

Beneficial effects of RBP modulation on CAR‐T‐cell activity have been proven in tumour contexts. Double depletion of Regnase‐1 and Roquin‐1 RBPs, normally limiting inflammatory responses in T‐cells, improves CAR‐T‐cells’ anti‐tumour functions [32]. Depletion of both Regnase‐1 RBP and BCL6 Corepressor factor increases the stemness of precursor exhausted T‐cells, and overcomes the epigenetic barriers that prevent restoration of exhausted T‐cell functions in chronic infections [33]. Moreover, depletion of Regnase‐1 and BCL6 Corepressor in anti‐CD19 CAR‐T‐cells confers them the capacity of self‐renewal without losing effector functions [34]. CAR‐T‐cell activity can also be enhanced through epigenetic reprogramming, as demonstrated by the case of a patient whose biallelic disruption of Ten Eleven Translocase (TET) 2 DNA demethylase in a clone of anti‐CD19 CAR‐T‐cells induced a memory phenotype, enhancing their tumour‐fighting capacity [35]. Metabolic rewiring also improves CAR‐T‐cell functions; for example, increased glucose availability re‐shapes CAR‐T‐cell metabolism and improves their functionality [36].

We hypothesise that similar manipulations of RBP‐mediated post‐transcriptional mechanisms, epigenetics and metabolism may also boost CAR‐T‐cells’ activity against auto‐antibody‐producing B‐cells, a hallmark of many autoimmune conditions. A deeper understanding of these processes and their interplay is therefore necessary to inspire new therapeutic strategies for autoimmunity.

## 2 Interplay of Epigenetic Factors and RBPs in Lymphocyte Nuclear RNA Processing

Early “post‐transcriptional” RNA processing steps, including splicing and mRNA cleavage and polyadenylation, take place co‐transcriptionally [37] and are regulated by the interplay of DNA/histone modifications, chromatin‐associated factors, and RBPs. RNA methylation is also co‐transcriptional [37], and its deposition can be guided by epigenetic modifications [38]. Here we focus on RNA splicing, cleavage, and polyadenylation with examples from T‐/B‐cells, while for an in‐depth discussion of the interconnection of epigenetics and the epitranscriptome, we direct the reader to specialised reviews [39, 40].

### 2.1 Alternative Splicing

**FIGURE 1:**
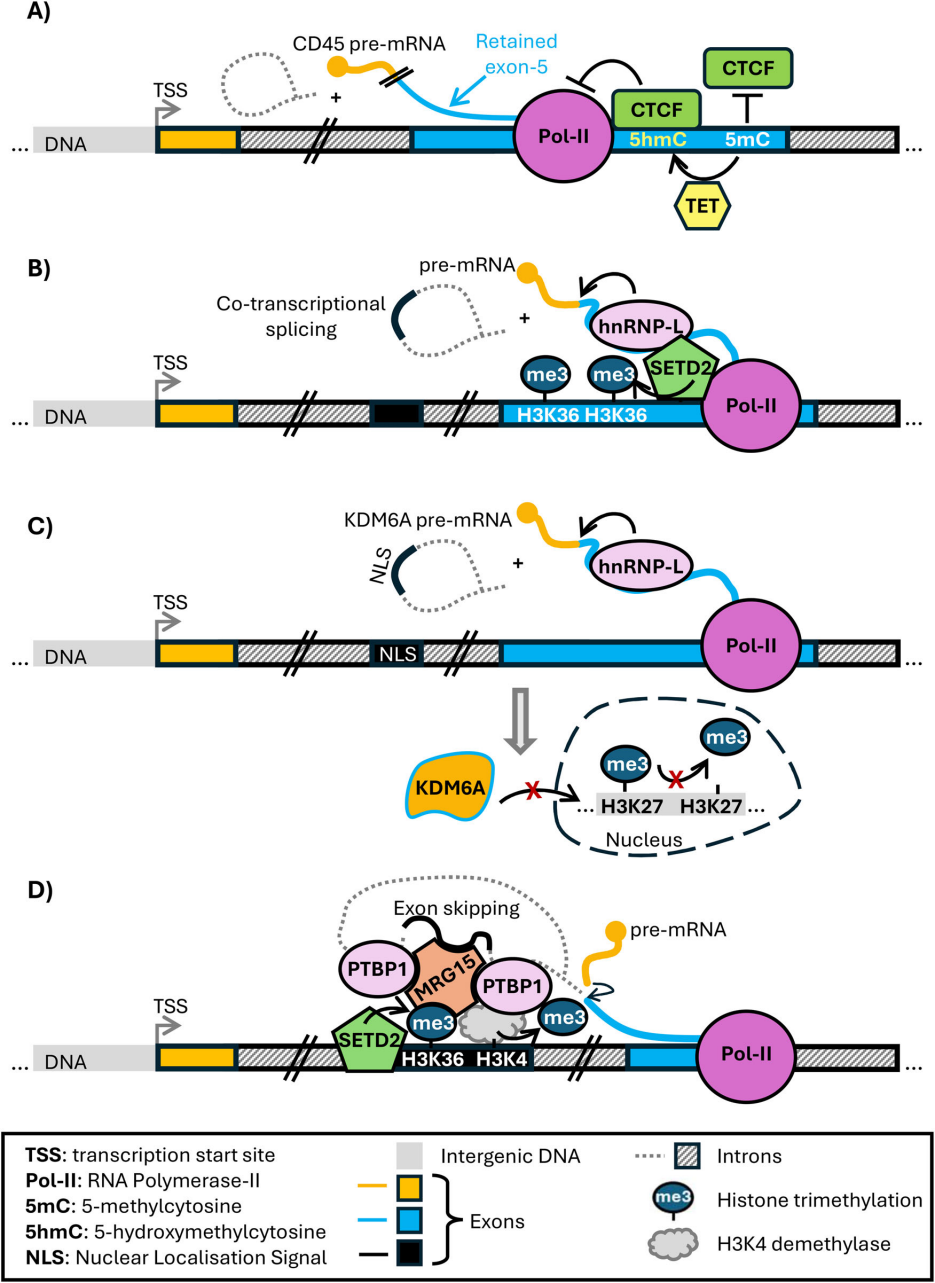
**(A)** DNA‐methylation and CTCF regulate CD45 exon‐5 AS [47, 48]. **(B)** HnRNP‐L/SETD2/Pol‐II axis regulates co‐transcriptional AS [51, 52]. **(C)** HnRNP‐L‐mediated KDM6A AS excludes an exon containing a nuclear localisation signal, leading to decreased KDM6A nuclear activity and increased histone methylation [53]. **(D)** Schematisation of the H3K36me3/MRG15‐mediated recruitment of PTBP1, which promotes exon skipping [59].

Chromatin organisation, nucleosome distribution and DNA/histone modifications modulate RNA Polymerase‐II (Pol‐II) elongation rate, affecting co‐transcriptional RNA processing events such as alternative splicing (AS) (reviewed in ref [41]). An early example of the interplay between epigenetic modifications, elongating Pol‐II, and splicing is the CCCTC‐binding factor (CTCF)‐dependent AS. Known mainly for its role in organising 3D chromatin architecture, CTCF also binds RNAs [42, 43, 44] and indirectly regulates splicing too [45]. For example, CTCF regulates AS of CD45 pre‐mRNA, which is expressed by all hematopoietic cells. Exons 4, 5 and 6 encode a region of the CD45 extracellular domain and undergo AS, leading to different CD45 isoforms whose expression is regulated during T‐cell maturation and activation [46]. CTCF binds exon‐5 DNA, causing Pol‐II to pause and allowing exon‐5 to be included in the nascent RNA [47] (Figure 1A). DNA methylation also has a role in modulating CTCF‐mediated AS, as 5mC inhibits CTCF binding to DNA [47]. A follow‐up study [48] clarified that when exon‐5 is retained, its DNA is enriched with 5‐hydroxymethylcytosine, a stable intermediate DNA modification produced during TET‐dependent demethylation. The authors showed that in activated CD4^+^ T‐cells, TET proteins are downregulated and CD45 exon‐5 is skipped [48]. Instead, in naïve CD4^+^ T‐cells, TET1 directly binds to exon‐5 to catalyse 5mC conversion to 5‐hydroxymethylcytosine, allowing CTCF binding and exon‐5 retention [48] (Figure 1A). Although, to our knowledge, CD45 exon‐5 splicing is not associated with disease, mutations leading to the aberrant inclusion of exon‐4 may be linked to autoimmune disorders [46].

As principal mediators of co‐transcriptional RNA processing, RBPs collaborate with epigenetic factors to regulate AS, and can also directly influence the epigenetic landscape. This is the case of the heterogeneous nuclear ribonucleoproteins (hnRNPs) hnRNP‐L and Polypyrimidine Tract Binding Protein 1 (PTBP1, or hnRNP‐I). The AS regulator hnRNP‐L is brought by SETD2 histone methyltransferase to specific chromatin *loci* where it mediates AS. SETD2 deposits H3K36me3 histone modification [49], which is enriched in exons [50]. It coordinates splicing by interacting with hnRNP‐L through its SETD2‐hnRNP interaction domain, and with elongating Pol‐II, acting as a bridge between the two [51] (Figure 1B).

Reciprocally, hnRNP‐L affects the epigenetic landscape. By binding to both nascent RNAs and SETD2, hnRNP‐L directs SETD2‐mediated H3K36me3 deposition at specific chromatin *loci* [52]. In support of this, hnRNP‐L knock‐down reduces H3K36me3 levels [52]. hnRNP‐L also affects AS of transcripts encoding epigenetic enzymes, as demonstrated in B‐cells. Here, hnRNP‐L loss deregulates AS, impairing B‐cell activation and antibody responses [53]. Amongst hnRNP‐L target transcripts whose AS is affected are mRNAs encoding SIRT1 histone deacetylase and KDM6A histone demethylase [53]. Following hnRNP‐L depletion, the inclusion of an exon containing a premature stop codon in SIRT1 mRNA behaves as a “poison” exon and represses SIRT1 expression [53]. Instead, the inclusion of a nuclear localisation signal in KDM6A mRNA increases KDM6A protein activity [53] (Figure 1C). These events trigger a cascade effect on gene expression mediated by further epigenetic remodelling [53].

hnRNP‐L also acts in thymocytes to support T‐cell development, at least in part by binding an exonic splicing silencer sequence in exon‐4 of CD45 transcript and mediating its skipping [54, 55]. Interestingly, a polymorphism disrupting this silencer sequence (and *bona fide* hnRNP‐L binding) increases exon‐4 inclusion in CD45 mRNA and is associated with multiple sclerosis [56]. Also, hnRNP‐L's partner SETD2 inhibits T‐helper (Th) 17 cell differentiation and promotes differentiation of induced regulatory T‐cells (Treg) [57], which are fundamental to suppress autoimmunity. Concordantly, SETD2 is downregulated in peripheral blood mononuclear cells from autoimmune patients [57]. Therefore, we could hypothesise that sustaining hnRNP‐L and SETD2 function could mitigate autoimmune reactions.

PTBP1 is an RBP which has a role in splicing, predominantly causing exon skipping [58]. SETD2‐deposited H3K36me3 is enriched on a subset of genes undergoing PTBP1‐dependent exon skipping. Here, H3K36me3 is bound by MRG15, which also binds the pre‐mRNA at the same time. MRG15 then recruits a H3K4me3 demethylase and PTBP1, which mediates exon skipping [59] (Figure 1D). Notably, PTPB1 can also affect the epigenetic landscape. For example, PTBP1 mediates the splicing of the DNA methyltransferase DNMT3B pre‐mRNA, leading to a functional DNMT3B protein during early mouse embryonic development. During neuronal differentiation, low PTBP1 levels cause retention of DNMT3B intron‐6 in the mature transcript, causing frameshift and degradation of the mRNA by nonsense‐mediated decay [60]. The consequent downregulation of DNMT3B could protect neuronal genes from DNA methylation and repression [60]. Interestingly, mutations in DNMT3B cause the Immunodeficiency Centromeric instability and Facial anomalies (ICF) type 1 syndrome, characterised by low immunoglobulin levels [61]. Also, DNMT3B‐mutant mouse models of ICF syndrome show thymocyte apoptosis after birth [62], although this is rare in ICF syndrome patients [61]. Future research may reveal mechanistic links between PTBP1‐mediated DNMT3B splicing and immunodeficiencies.

In mice, PTBP1 regulates B‐cell development, maintenance, and proliferation in the germinal centre, sometimes redundantly with other PTB proteins [63, 64, 65]. PTBP1 contributes to the activation of naïve CD8^+^ T‐cells, in part by regulating AS of calcineurin Aβ, a factor involved in T‐cell receptor signalling [66]. Given its important role in B‐ and T‐cell biology, PTBP1 has been implicated in autoimmunity [67]. Indeed, PTBP1 contributes to immune adaptation in the colon, as mice in which PTBP1 was specifically depleted from intestinal epithelial cells develop colitis and early colorectal cancer [68].

A further example in which a histone modification guides RBP‐mediated AS is the H3K36me3/PSIP1/SRSF1 axis. H3K36me3 recruits the serine‐arginine‐rich splicing factor SRSF1 to specific chromatin *loci via* the short isoform (p52) of PC4‐ and SF2‐Interacting Protein‐1 (PSIP1). PSIP1 interacts with H3K36me3 and recruits SRSF1; therefore, its depletion affects SRSF1‐mediated splicing [69]. Both PSIP1 and SRSF1 sustain T‐lymphocyte homeostasis and functions. PSIP1 is a chromatin factor that binds L‐arginine and mediates L‐arginine‐induced T‐cell survival [70]. SRSF1 controls distinct pathways in mouse regulatory and effector CD4^+^ T‐cells (e.g., differentiation, homeostasis and cytokine production) [71], and in effector CD8^+^ T‐cells, SRSF1 contributes to the exclusion of a destabilising “poison” exon from the mRNA encoding the splicing factor TRA2B, whose function is fundamental for T‐cell activation [11]. Therefore, we hypothesise that increasing PSIP1 and/or SRSF1 expression could improve the outcome of engineered cell‐based therapies.

SRSF1 also protects against autoimmunity. Indeed, SRSF1 expression in Treg sustains their survival and functions [72]. Total T‐lymphocytes from the peripheral blood of patients with systemic lupus erythematosus (SLE) show decreased SRSF1 expression [73, 74], and T‐lymphocyte‐specific SRSF1 deficiency in mice induces lupus‐like autoimmunity [75] and lymphopenia [76]. Further investigation is needed to clarify the T‐cell subtypes through which SRSF1 downregulation mediates these autoimmune symptoms. This could open the way to a viable strategy to treat autoimmune conditions *via* modulating SRSF1 expression in specific T‐cell populations.

At a more general level, the significance of the interplay between DNA/histone modifications and RBP‐mediated splicing is confirmed by the discovery that combinations of histone PTMs and DNA methylation in human cell lines establish “chromatin signatures” promoting similar AS events, and each signature marks exons belonging to genes involved in similar pathways [77]. Some chromatin signatures are also enriched for AS‐related RBP‐binding motifs on the alternatively spliced exons or flanking introns [77]. Both epigenetic marks and RBP‐binding motifs contribute to the recruitment of specific AS regulators to chromatin. Changes in chromatin signature on alternatively spliced exons can influence the recruitment of RBPs, leading to different AS events [77]. This finding corroborates the strong collaboration between epigenetic modifications and RBPs during splicing to regulate transcript isoforms and gene expression.

### 2.2 mRNA Cleavage and Polyadenylation

**FIGURE 2:**
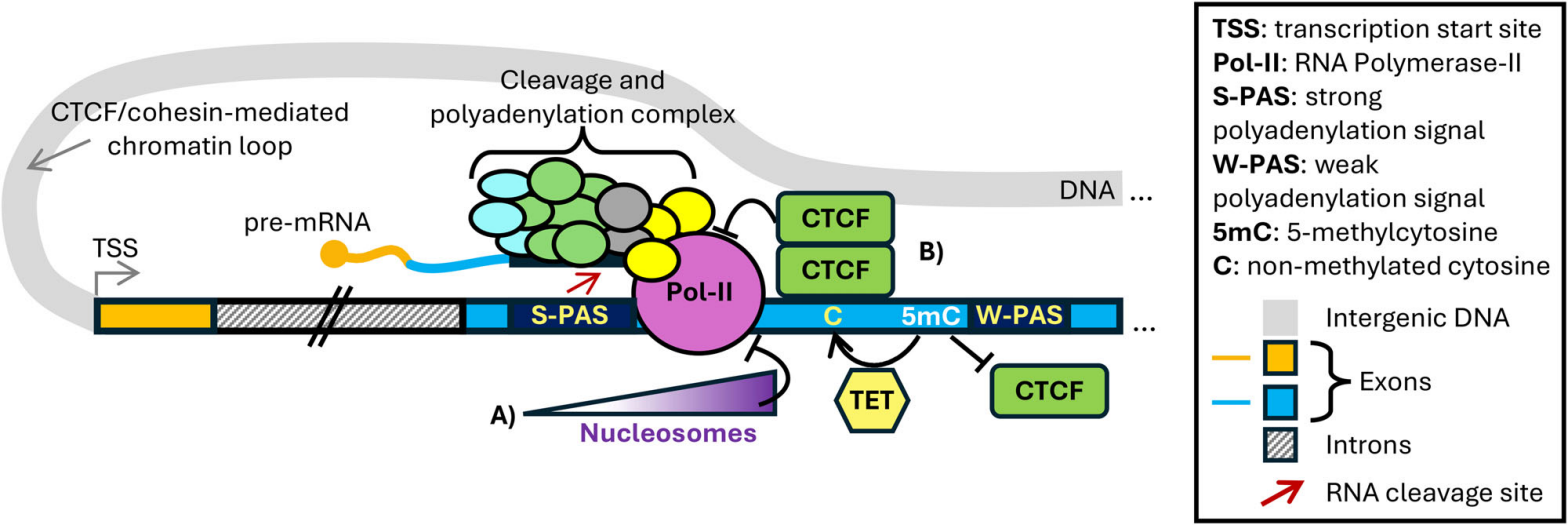
This figure merges different epigenetic mechanisms regulating APA choice: **(A)** nucleosome density around a strong PAS [81]; **(B)** methylation status of CpG islands between two APAs and consequent regulation of CTCF recruitment and chromatin loop formation [85].

Cleavage and polyadenylation of nascent transcripts is a co‐transcriptional process dependent on in‐*cis* sequences in the polyadenylation signal (PAS) at the 3′ end of the pre‐mRNA and subunits of the cleavage and polyadenylation (CPA) complex. An endonuclease in the CPA complex cleaves the transcript, and the upstream 3′ end of the RNA is polyadenylated [78]. AS can determine the availability of alternative PASs (APAs) leading to alternative 3′ untranslated regions (UTRs) or protein isoforms [79]. As for splicing, Pol‐II elongation rate influences CPA complex binding and affects APA choice. A Pol‐II that slows over the PAS favours CPA complex association and transcription termination (reviewed in ref [80]). Chromatin structure and DNA/histone modifications regulate Pol‐II elongation rate. Indeed, in human genes, nucleosomes are strongly depleted from preferred PASs around the AATAAA motif, while they are enriched in a window from 75 to 375 bp downstream of the PAS [81], possibly slowing down the polymerase (Figure 2A). 5mC can also regulate APA: a classic example from mice is the imprinted minor histocompatibility locus H13, relevant for transplant rejection [82, 83]. In the H13 gene, a CpG island is located between two sets of APAs, one used by the maternal allele and the other by the paternal allele [84]. When the CpG island is methylated (maternal allele), distal PASs are preferred and *vice versa* [84]. This may correlate with the effect that DNA methylation has on the transcriptional activity of a promoter found at the level of the CpG island [84]. Another APA mechanism involves CpG islands located between two APAs, which, when unmethylated, bind CTCF. CTCF recruits the cohesin complex, which generates a chromatin loop that interferes with transcription elongation and favours the use of the proximal APA [85] (Figure 2B).

Sequence‐specific RBPs regulate cleavage and polyadenylation, firstly because CPA complex proteins bind pre‐mRNA. Secondly, RBPs regulating AS can also affect APA when these two processes are linked. In addition, some RBPs, including MBNL [86] or FUS [87], regulate APA use through recruitment of the CPA complex, depending on the location of the RBP binding site relative to the PAS [88]. The role of RBPs in regulating polyadenylation was previously reviewed by others [89, 90, 91]. RBPs regulating PAS choice can also be implicated in inflammation. This is the case of the RBPs HuB and HuC, which contribute to maintaining long 3′UTRs in the human neuronal transcriptome [92], possibly by inhibiting the recognition of proximal PAS as in Drosophila [93]. Long 3′UTRs can adopt double‐stranded secondary structures which stimulate inflammatory pathways and anti‐viral responses in neurons [92]. However, excessive amounts of double‐stranded RNA can lead to neuroinflammation, as happens in several neurodegenerative and autoimmune diseases [92]. In lymphocytes, the choice of APAs is regulated, and disruption of PAS can lead to autoimmune diseases. APAs are differentially chosen in naïve *versus* activated T‐cells. For example, naïve T‐cells display low levels of CSTF2 (a CPA complex subunit), so they cannot use the weaker proximal APA in the NF‐ATc pre‐mRNA [94]. Instead, splicing occurs, and the strong distal APA is used [94]. By contrast, in activated T‐cells, CSTF2 levels are higher, and the proximal APA can be used [94]. At a broader level, it has been shown that in activated mouse CD4^+^ T‐cells, there is a global preference for using upstream APAs, leading to short 3′UTRs [95]. Subsequent work clarified that changes in APA usage during CD4^+^ T‐cell activation also result in lengthening events and are transient during clonal expansion [96]. Although 3′UTR shortening may allow mRNAs to escape targeting by microRNAs and RBPs, this has only limited effects on RNA/protein levels in activated T‐cells [97]. However, the absence of a global effect does not diminish the importance that individual cases of 3’UTR length regulation may have on T‐cell activation. In autoimmune diseases, cleavage and polyadenylation can be altered by mutations occurring inside the PAS. For instance, an A–to‐G transition disrupts the main PAS of FOXP3, leading to a longer and less stable transcript. This mutation was proposed to cause the rare immunodysregulation–polyendocrinopathy–enteropathy X‐linked autoimmune syndrome [98].

## 3 Interconnection of Metabolic, Post‐Transcriptional and Epigenetic Processes in Immunity

In this section, which places emphasis on lymphocyte biology, we consider the relationship between metabolism and epigenetics and the importance of RBPs in these processes.

### 3.1 Metabolites as Substrates for Epigenetic Enzymes

Cellular metabolites act as substrates for and regulators of chromatin modifiers. Three well‐known examples, which we will focus on below, are (1) SAM, an intermediate of the methionine cycle and substrate for DNA/histone methyltransferases, (2) α‐ketoglutarate, produced within the tricarboxylic acid cycle and glutamine metabolism and substrate for DNA/histone demethylates, (3) acetyl‐CoA, substrate for histone acetyltransferases, whose cytoplasmic and nuclear levels are regulated by different pathways including glycolysis and fatty acid oxidation [19]. These metabolites regulate the balance between DNA/histone methylation/demethylation and histone acetylation levels, respectively, impacting chromatin accessibility and gene expression [19].

### 3.2 Metabolism‐Dependent Epigenetic Regulation in T‐Cells

The relationship between metabolism, epigenetics and gene expression is widely studied in lymphocytes, particularly T‐cells. Its relevance lies in its potential to inspire therapeutic interventions to modulate immune responses in cancer and autoimmunity.

SAM availability and DNA/histone methylation have been shown to affect T‐cell differentiation and effector functions. During differentiation, Th1 and Th17 cells increase uptake of extracellular methionine, methionine metabolism and SAM production compared with naïve cells [99]. SAM is then used as a substrate by histone methyltransferases. In Th17 cells, global H3K4me3 deposition is particularly sensitive to extracellular methionine. Upon methionine restriction, reduction of H3K4me3 at promoters is coupled to decreased expression of many proliferation‐related genes [99]. Methionine restriction also affects Th effector functions, decreasing production of IL‐17 in Th‐17 and of IFNγ in Th1 [99]. Given the critical role of pathogenic Th cells in sustaining autoimmunity [100], limiting methionine availability, for example by following a methionine‐low diet, may offer therapeutic opportunities for T‐cell‐mediated autoimmune diseases. Indeed, a methionine‐restricted diet reduces immune infiltration in the central nervous system of mice with experimental autoimmune encephalomyelitis [99], a model of autoimmunity in the central nervous system.

The activity of α‐ketoglutarate‐dependent demethylases in T‐cells is also profoundly affected by metabolism. For example, an enantiomer of 2‐hydroxyglutarate (R‐2‐HG) inhibits α‐ketoglutarate‐dependent histone lysine‐demethylases [101]. CD8^+^ T‐cells treated with R‐2‐HG show increased levels of H3K27me3 and reduced activation [101]. Instead, another enantiomer of 2‐hydroxyglutarate (S‐2‐HG) has limited effects on α‐ketoglutarate‐dependent demethylases, and CD8^+^ T‐cells treated with it show increased proliferation, memory and effector gene signatures, and anti‐tumour functions [101]. Treatment with S‐2‐HG could therefore boost CAR‐T‐cell activity in both cancer and autoimmune settings.

Regulation of acetyl‐CoA production, and consequently of histone acetylation, is another well‐studied link between metabolism and epigenetics in T‐cells. Acetyl‐CoA produced by the pyruvate dehydrogenase PDH has a fundamental role in CD4^+^ T‐cell activation, and following T‐cell receptor stimulation, PDH itself enters the nucleus and binds to an acetyltransferase and to acetylated histones, possibly contributing to the generation of acetyl‐CoA for histone acetylation at the chromatin level [102].

During activation, T‐cells reprogramme their metabolism by increasing aerobic glycolysis [103]. Activated T‐cells, particularly Th17, express the glucose transporter GLUT3. In Th17, GLUT3 fuels aerobic glycolysis and potentiates the tricarboxylic acid cycle and ATP Citrate Lyase (ACLY)‐mediated generation of acetyl‐CoA [104]. In the nucleus, acetyl‐CoA is a substrate for histone acetylation at Th17‐signature genes, increasing their expression [104]. Ectopic GLUT3 expression in Th17 supports IL‐17A and GM‐CSF expression *in vitro* and worsens Th17‐mediated autoimmunity *in vivo*, while T‐cell‐specific GLUT3 knock‐out prevents autoimmunity in mouse models of experimental autoimmune encephalomyelitis and colitis [104].

The metabolic source of nuclear acetyl‐CoA can determine T‐cell responses, as in the case of chronically stimulated CD8^+^ T‐cells [105]. Acetate‐derived acetyl‐CoA is produced by Acyl‐CoA Synthetase Short‐chain family member‐2 (ACSS2), while citrate‐derived acetyl‐CoA is produced by ACLY. Exhausted T‐cells downregulate ACSS2 but not ACLY, reducing the production of acetyl‐CoA from acetate [105]. In the nucleus, ACLY and ACSS2 interact with KAT2A and p300 histone acetyltransferases, respectively. The two complexes acetylate histones at different *loci*, with ACLY/KAT2A acetylating genes associated with terminal exhaustion, likely increasing their expression, and ACSS2/p300 acetylating effector and memory genes [105]. The authors showed that ACLY inhibition induces ACSS2 expression *in vitro* and increases T‐cell anti‐tumour activity in tumour‐bearing mice, synergising with immune checkpoint blockade treatment [105]. This suggests new strategies to reduce exhaustion in tumours or chronic infections.

### 3.3 RBPs as Post‐Transcriptional Mediators of Immune Metabolism and Epigenetics

RBPs modulate metabolic regulation of epigenetics by targeting transcripts encoding metabolic enzymes, both in the nucleus and in the cytoplasm. For example, PTBP1 contributes to the choice between the mutually exclusive exons 9 and 10 in M‐type‐pyruvate kinase (PKM) transcript. By promoting exon‐9 exclusion and exon‐10 inclusion, PTBP1 favours the expression of the PKM2 isoform, which supports aerobic glycolysis and the anabolic processes needed for cell growth and division [23]. By contrast, exon‐9 inclusion generates the PKM1 isoform, which sustains oxidative phosphorylation [23]. This is an example of RBP‐mediated metabolic switching, which can regulate the availability of substrates/cofactors for epigenetic regulators. In the cytoplasm, the RBPs Roquin‐1 and Roquin‐2 destabilise the mRNA encoding the iron transporter Transferrin receptor‐1 in iron‐rich conditions, by binding to secondary structures in its 3’UTR [106]. Therefore, Roquin‐1/2 modulation could affect iron uptake and the activity of Fe^2+^‐dependent enzymes such as Jumonji‐C histone‐ and TET DNA‐demethylases.

Another RBP regulating a metabolic enzyme which impacts the epigenetic landscape is RNA‐binding motif protein 25 (RBM25). RBM25 binds to ACLY pre‐mRNA and favours exon‐14 inclusion; in contrast, RBM25 depletion induces exon‐14 skipping, leading to the accumulation of a short ACLY isoform [22]. Because ACLY activity is decreased by lactylation, which is dependent on an amino acid encoded by exon‐14, in RBM25‐depleted macrophages a more active unlactylated ACLY accumulates, resulting in higher citrate‐derived acetyl‐CoA production, increased histone acetylation and higher expression of proinflammatory genes [22]. Mice with macrophage‐specific RBM25 depletion develop severe arthritis symptoms after immunisation with type‐II collagen [22]. Interestingly, RBM25 RNA and protein levels are also decreased in synovial tissues from rheumatoid arthritis patients [22]. Increasing its expression could represent a therapeutic opportunity to alleviate autoimmune symptoms.

**FIGURE 3:**
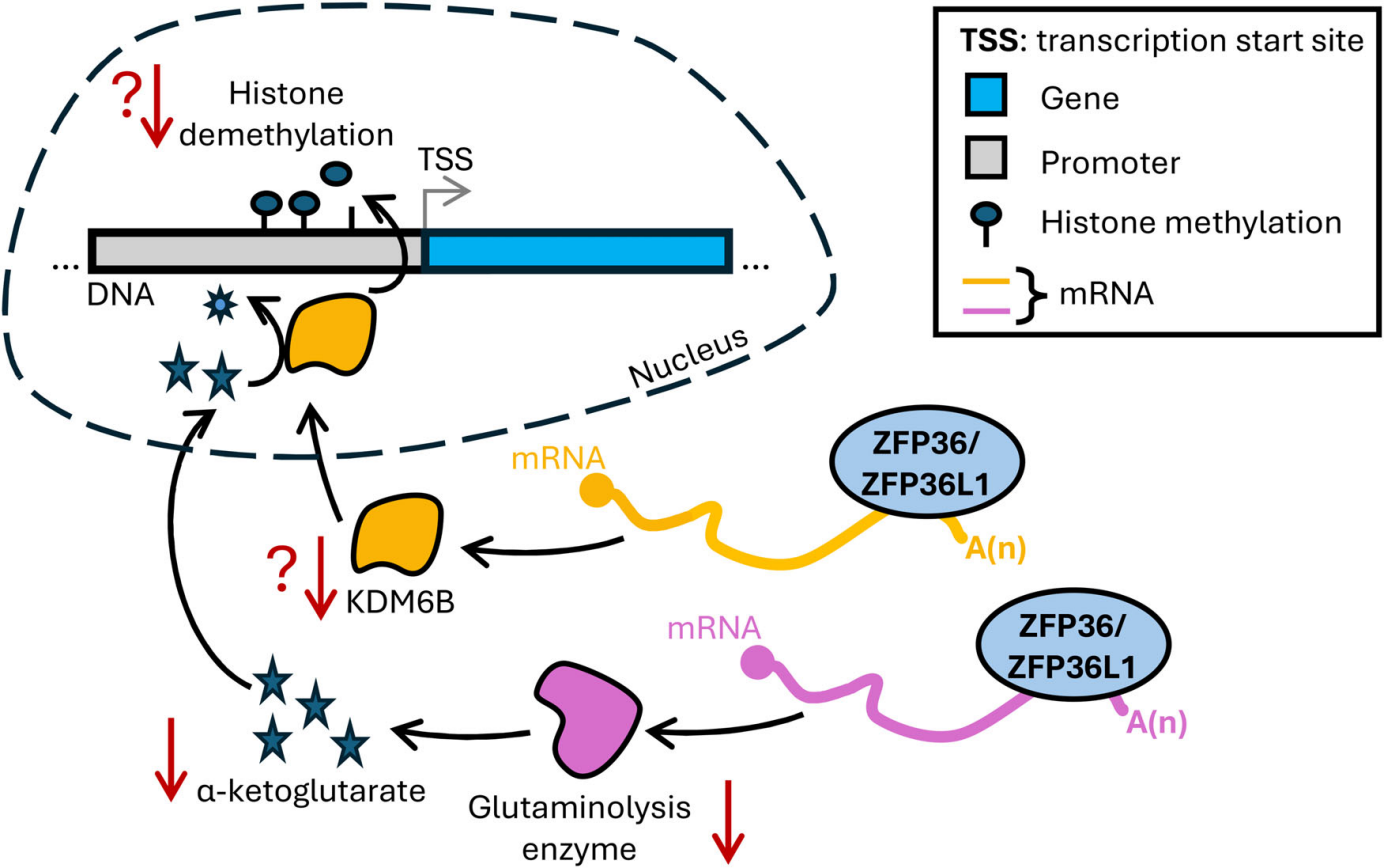
ZFP36‐family RBPs target mRNAs encoding glutaminolysis enzymes, leading to decreased levels of α‐ketoglutarate [21], and KDM6B mRNA [117] encoding a histone demethylase using α‐ketoglutarate as a substrate, probably decreasing its levels. These two mechanisms may converge in the nucleus, resulting in increased histone methylation levels.

In addition to metabolic mRNAs, RBPs also directly target transcripts encoding epigenetic enzymes. This is the case of the ZFP36‐family RBPs (including ZFP36, ZFP36L1, ZFP36L2), which are emerging as a link between metabolism [21, 107] and epigenetic regulation. They are known regulators of mRNA decay and inhibitors of translation [108] with a fundamental role in immunity [109, 110, 111, 112, 113, 114]. In CD4^+^ T‐cells, ZFP36/ZFP36L1 limit anabolic metabolism by directly targeting mRNAs encoding both metabolism‐related transcription factors and rate‐limiting metabolic enzymes [21]. Among the pathways limited by the ZFP36 family were glutaminolysis and α‐ketoglutarate production [21]. As α‐ketoglutarate is used as a substrate in DNA/RNA and histone demethylation reactions [19, 115], we hypothesise that ZFP36/ZFP36L1‐mediated repression of glutamine metabolism may affect the histone and DNA methylation landscape not only in CD4^+^ T‐cells, but also in other immune cells depending on ZFP36‐family proteins (e.g. splenic marginal zone B‐cells [116]). Interestingly, ZFP36/ZFP36L1 also target the mRNA encoding the histone demethylase KDM6B [117], which uses α‐ketoglutarate as a substrate and sustains CD8^+^ T‐cell activation, memory and effector functions [118, 119]. Although the effects of ZFP36/ZFP36L1 binding to KDM6B mRNA require further study, we suggest that ZFP36/ZFP36L1 limit KDM6B expression, given their role as negative regulators of transcript stability and translation. It is noteworthy that the reduction of both glutaminolysis and KDM6A/B improves autoimmune conditions. Glutaminolysis is fundamental for T‐cell activation, and its limitation alleviates symptoms of several autoimmune diseases in mouse models [120]. KDM6A/B inhibition reduces expression of interferon‐stimulated genes in monocytes from SLE patients and alleviates symptoms in a SLE mouse model [5]. Also, KDM6A/B inhibitor GSK‐J4 reshapes Th17 metabolism and has an anti‐inflammatory effect on these cells [121]. Therefore, we hypothesise that ZFP36/ZFP36L1 act as a hub to regulate metabolism, epigenetics and transcription factor activity through the limitation of glutaminolysis and the negative regulation of KDM6B activity (Figure 3). Sustaining ZFP36/ZFP36L1 expression in immune cells may protect from autoimmunity through the limitation of metabolic and epigenetic pathways supporting inflammation.

## 4 Conclusion

**FIGURE 4:**
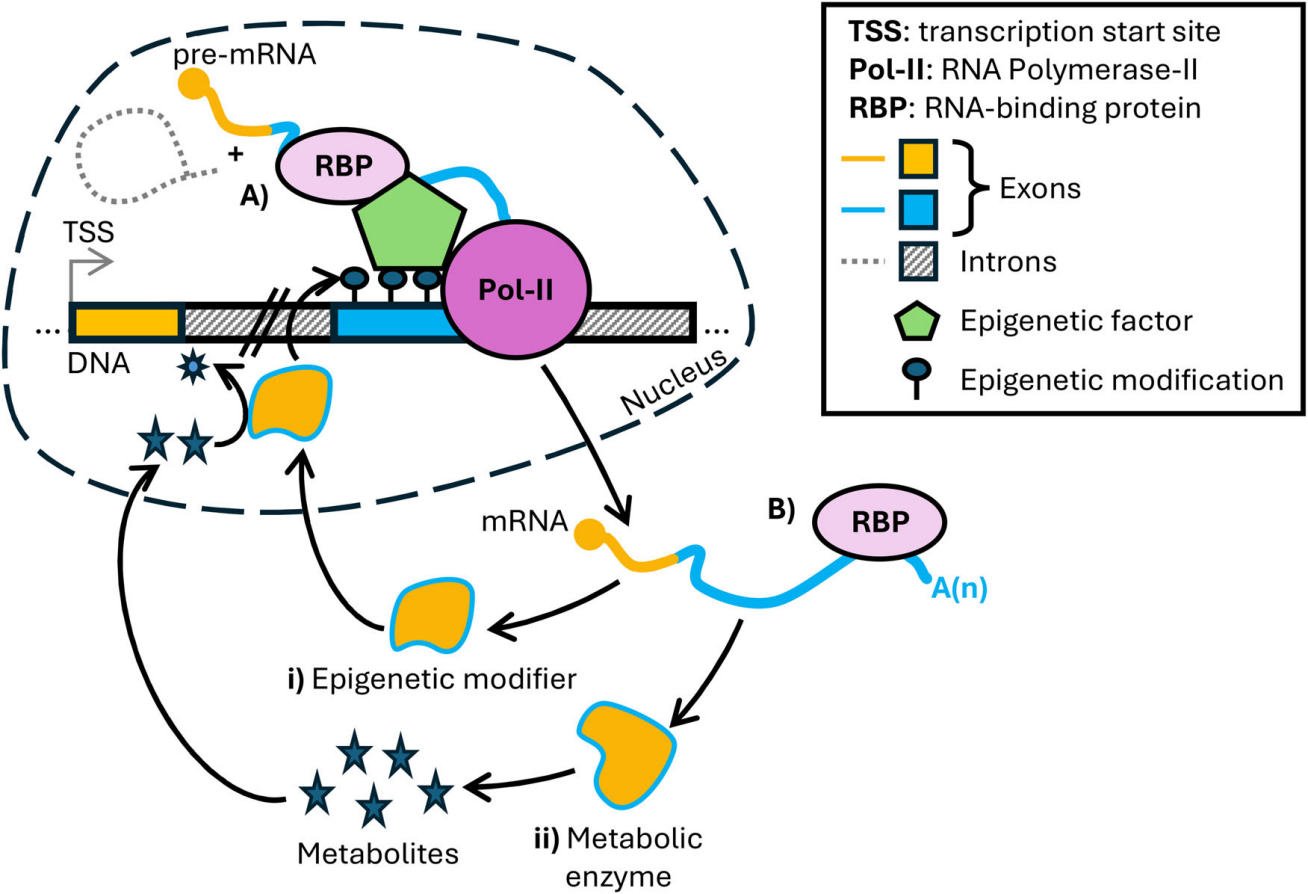
This figure summarises the interplay of epigenetics, metabolism and RBPs across the nucleus and cytoplasm. **(A)** In the nucleus, RBP‐mediated co‐transcriptional RNA processing and epigenetic mechanisms collaborate to regulate gene expression. In some cases, this interplay affects the expression of epigenetic (i) or metabolic (ii) factors. **(B)** In the cytoplasm, RBPs (e.g., ZFP36‐family proteins [21, 117]) target mRNAs encoding epigenetic (i) or metabolic (ii) factors, reinforcing the regulatory loop between post‐transcriptional regulation, metabolism and epigenetics.

Epigenetic processes and metabolism collaborate to integrate environmental and genetic inputs to regulate gene expression, with RBPs acting as “bridges” between post‐transcriptional RNA processing, epigenetic and metabolic reactions (Figure 4). In addition to those with known RNA‐binding domains or part of ribonucleoprotein complexes [122], many “non‐canonical” RBPs have been identified [123, 124] and new binding properties have been discovered for some DNA‐ and RNA‐binding proteins (i.e., DNA/RNA‐binding proteins [DRBPs]) [125, 126]. These discoveries delineate an additional level of complexity in the interconnection of transcription, epigenetics and post‐transcriptional regulation. Regarding DRBPs, in a recent effort to build a comprehensive RBP interaction map, ZNF800 and QKI have been found (and confirmed, respectively) to be chromatin‐associated RBPs regulating gene expression both transcriptionally and post‐transcriptionally [127]. Also, many zinc‐finger proteins are predisposed to bind both RNA and DNA, suggesting that they can bind DNA and co‐transcriptionally associate with their target RNAs [128]. The functional roles of DRBPs in T‐cell differentiation have been recently reviewed [129].

Although the direct effects of metabolism on co‐transcriptional events (e.g., splicing) are currently being revealed [130], and there are examples of RBP‐mediated cytoplasmic regulation of epigenetic and metabolic factors, we believe that there is great potential for further study of these processes, particularly in lymphocytes. Increasing our knowledge of these pathways will expand the range of targetable molecular events to regulate immune response and adoptive cell therapies against cancer and autoimmune diseases.

## Author Contributions

Francesca Rossi: research, writing—first draft and editing. Martin Turner: research, writing—review and editing.

## Conflicts of Interest

The authors declare no conflicts of interest. Some research in M. T.’s lab is funded by AstraZeneca.

## Peer Review

The peer review history for this article is available at https://publons.com/publon/10.1002/eji.70064.

## Permission to Reproduce Material from Other Sources

Not Applicable.

## Data Availability

Data sharing does not apply to this article as no datasets were generated or analysed during the current study.
